# RNA-Seq analysis reveals that multiple phytohormone biosynthesis and signal transduction pathways are reprogrammed in *curled-cotyledons* mutant of soybean [*Glycine max* (L.) Merr.]

**DOI:** 10.1186/1471-2164-15-510

**Published:** 2014-06-21

**Authors:** Guixia Shi, Fang Huang, Yu Gong, Guangli Xu, Jingjing Yu, Zhenbin Hu, Qingsheng Cai, Deyue Yu

**Affiliations:** College of Life Sciences, Nanjing Agricultural University, Nanjing, China; National Center for Soybean Improvement, National Key Laboratory of Crop Genetics and Germplasm Enhancement, Nanjing Agricultural University, Nanjing, 210095 China

**Keywords:** Soybean, RNA-seq, Plant hormone, Curled-cotyledon, Mutant

## Abstract

**Background:**

Soybean is one of the most economically important crops in the world. The cotyledon is the nutrient storage area in seeds, and it is critical for seed quality and yield. Cotyledon mutants are important for the genetic dissection of embryo patterning and seed development. However, the molecular mechanisms underlying soybean cotyledon development are largely unexplored.

**Results:**

In this study, we characterised a soybean ***c****urled-****co****tyledon* (*cco*) mutant. Compared with wild-type (WT), anatomical analysis revealed that the *cco* cotyledons at the torpedo stage became more slender and grew outward. The entire embryos of *cco* mutant resembled the “*tail of swallow*”. In addition, *cco* seeds displayed reduced germination rate and gibberellic acid (GA_3_) level, whereas the abscisic acid (ABA) and auxin (IAA) levels were increased. RNA-seq identified 1,093 differentially expressed genes (DEGs) between WT and the *cco* mutant. The KEGG pathway analysis showed many DEGs were mapped to the hormone biosynthesis and signal transduction pathways. Consistent with assays of hormones in seeds, the results of RNA-seq indicated auxin and ABA biosynthesis and signal transduction in *cco* were more active than in WT, while an early step in GA biosynthesis was blocked, as well as conversion rate of inactive GAs to bioactive GAs in GA signaling. Furthermore, genes participated in other hormone biosynthesis and signalling pathways such as cytokinin (CK), ethylene (ET), brassinosteroid (BR), and jasmonate acid (JA) were also affected in the *cco* mutant.

**Conclusions:**

Our data suggest that multiple phytohormone biosynthesis and signal transduction pathways are reprogrammed in *cco*, and changes in these pathways may partially contribute to the *cco* mutant phenotype, suggesting the involvement of multiple hormones in the coordination of soybean cotyledon development.

**Electronic supplementary material:**

The online version of this article (doi:10.1186/1471-2164-15-510) contains supplementary material, which is available to authorized users.

## Background

Cotyledon is the main nutrient storage area in soybean seed, which contains approximately 40% protein and 20% oil at maturity [1]. Flowering plants are divided into monocots and dicots based on the number of cotyledons. In *Arabidopsis*, as a paradigm for dicot embryonic development, cotyledons are specified from two lateral domains at the apical end of the embryo proper at the heart stage, showing bilateral symmetry [2]. Several studies have reported cotyledon mutants in *Arabidopsis* [3–7], *Antirrhinum* [8], tomato [9–11], and other plants [12]. These mutants have been used to identify a number of orthologous gene hierarchies that are involved in cotyledon development [13]. The *Arabidopsis cup-shaped cotyledon* (*CUC*) genes (*CUC1*, *CUC2*, and *CUC3*), belonging to the plant-specific NAC transcription factor, *Picea glauca NAC01*, and *SHOOT MERISTEMLESS* (*STM*) are required for shoot apical meristem (SAM) formation and cotyledon separation [4, 14–16]. *STM* induces the expression of *CUC* genes, and the induction is specific and independent of other meristem regulators [17]. *STM* negatively regulates *ASYMMETRIC LEAVES1* (*AS1*), and the mutation of *AS1* in *Arabidopsis* disrupts cotyledon development [18]. *KNOTTED*-like (*KNAT6)* is expressed at the boundary between the SAM and cotyledon later than *STM* and *CUC*, suggesting that *KNAT6* plays a crucial role in SAM maintenance and boundary establishment in embryos via the *STM/CUC* pathway [19].

Phytohormones, primarily auxin, CK, BR, GA, ABA, and JA, orchestrate intrinsic developmental programs. Previous studies have greatly advanced the functions of individual hormone. During last two decades, extensive lists of genes involved in hormone synthesis, catabolism and signal transduction have been identified via the analysis of mutants [20–23]. Auxin plays a key regulatory role in the initiation of cotyledon in the apical margin of the globular embryo [24]. In many plant species, including *Brassica napus* [25], *Arabidopsis* [26], and *Norway spruce* [27], studies have shown that cotyledon and SAM development could be inhibited by various polar auxin inhibitors in embryogenesis. PIN auxin efflux regulators are the best characterised components of auxin transport. The polar location of PIN1 is associated with the position of auxin accumulation in incipient cotyledons, and auxin flows into the primordium interior during cotyledon outgrowth [28]. The *pin1 pid* double mutant completely lacks cotyledons and bilateral symmetry and exhibits increased *CUC1*, *CUC2* and *STM* expression, indicating that directional auxin transport is important for the establishment of bilateral symmetry and the promotion of cotyledon outgrowth [26]. Similarly, the *pid enp* double mutant, which is similar to *“laterne”*, also abolishes cotyledon development, reflecting failed auxin accumulation in the apex, and *ENP* is necessary for cotyledon development via the control of *PIN1* polarity in the context of *PID* [29]. In addition to auxin, some studies have shown that CK affects cotyledon development. Exogenous application of benzyladenine (the synthetic cytokinin) onto hybrid larch somatic embryos reduced the cotyledon number [30]. The *amp*-*arabidopsis* mutant demonstrated polycotyly, indicating an elevated cytokinin level [31]. *Arabidopsis ESR1* and *ESR2*, whose expression levels are induced by cytokinin, not only control shoot regeneration but also play a role in cotyledon development [32, 33].

In this study, we characterised a soybean mutant named *cco*, which displayed curled cotyledons. The *cco* embryos at the torpedo stage resembled the “*tail of swallow*”. High performance liquid chromatography (HPLC) analysis showed that *cco* seeds contained higher IAA and ABA levels, but lower GA_3_ level. Transcriptome analysis revealed that multiple phytohormone biosynthesis and signal transduction pathways were reprogrammed in *cco*.

## Results and discussion

### Embryogenesis in the *cco*mutant

**Figure 1:**
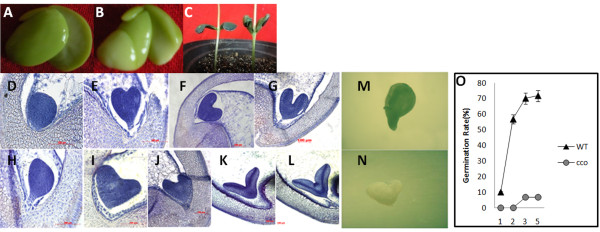
**Embryogenesis of the** ***cco*** **mutant. (A)** Cotyledons of WT. **(B)** Cotyledons of the *cco* mutant. **(C)** Cotyledons after germination for 5 days. WT (left) and the *cco* mutant (right). **(D)** WT embryo at the globular stage approximately 9 days after fertilisation. **(E)** WT embryo at the heart stage approximately 11 days after fertilisation. **(F)** and **(G)** WT embryo at the torpedo stage approximately 13 days after fertilisation. **(H)** *cco* embryo at the globular stage approximately 11 days after fertilisation. **(I)** *cco* embryo at the heart stage approximately 13 days after fertilisation. **(J)** to **(L)** *cco* embryos at the different torpedo stages approximately 15 days after fertilisation. Bar = 100 μm for **(D)** to **(L)**. **(M)** WT embryo at the cotyledon stage. **(N)** *cco* embryo at the cotyledon stage. **(O)** Germination rates of WT and *cco*. WT, triangles; *cco*, circles. **P < 0.01.

We obtained the *cco* mutant following sodium azide (NaN_3_) and ^60^Coγ ray seed mutagenesis of soybean [34], which was initially characterised by outward folding cotyledons (Figure 1A-C, 1M and 1N). Compared with WT, developing cotyledons of *cco* mutant had larger vacuoles and more membranous multilamellar structures, as well as higher methionine and cysteine content [34]. In addition, the germination rates of seeds were compared between the *cco* mutant and WT. In general, seeds from WT germinated on the second day, and the germination rate reached ~72% on the fifth day. *cco* seeds were dormant until the third day, and the germination rate was only ~6.7% on fifth day, suggesting that the germination rate of *cco* seeds was greatly reduced (Figure 1O).

To determine the embryogenesis defects in *cco* mutant, we traced its phenotype back to the globular stage of embryogenesis. In higher plants, embryogenesis can be conceptually divided into three overlapping phases: morphogenesis, maturation and desiccation [35]. In general, for the wild type at the globular stage, an embryo comprises a spherical embryo proper and a suspensor (Figure 1D). The embryo proper is spherical during the pro-embryo and globular stage and eventually forms cotyledons, shoot meristem and hypocotyl region around the apical-basal axis, and this structure is connected to the embryo sac via the suspensor. During the globular-heart transition phase, a dramatic change occurs: two of the cotyledon primordia are symmetrically initiated from the lateral domains at the apical end of the heart-shaped embryo proper (Figure 1E). Subsequently, the cotyledons and hypocotyl elongate, and the embryo enters the torpedo stage in which the cotyledons continue to rotate. The morphogenesis phase of embryogenesis ends at the cotyledon stage (Figure 1F and 1G).

However, the development of embryos in *cco* was slower than that in WT (Figure 1). When WT embryos were at the heart stage, *cco* embryos remained morphologically at the globular stage (Figure 1E and 1H). The embryo proper in *cco* appeared smaller than that in WT (Figure 1D and 1H). At the heart stage, cotyledons were initiated from the cotyledon primordia. Figure 1E and 1I showed that the cotyledon initiation positions were identical in both the WT and *cco*. However, the WT cotyledons grew upwards, while that of the *cco* mutant demonstrated outward growth. Subsequently, the bifurcation of the cotyledons at the torpedo stage in the *cco* mutant was larger, and the tips of the cotyledons were sharper (Figure 1F to 1G and 1J to 1L). At the torpedo stage, *cco* mutant embryos resembled “*tail of swallow*”, while the radicle and shoot apical meristem were also present and appeared normal. All in all, *cco* showed abnormal embryogenesis, especially at the torpedo stage, when the *cco* cotyledons became more slender and grew outward. However, SAM and the polar axis were normal. Cotyledons and leaves share homology in plants [13]. Cotyledon initiation and development in *cco* were defective, while leaf development was correct, suggesting partial homology between leaf and cotyledon.

### Increased free IAA and ABA levels, while decreased GA_3_level in *cco*

**Figure 2:**
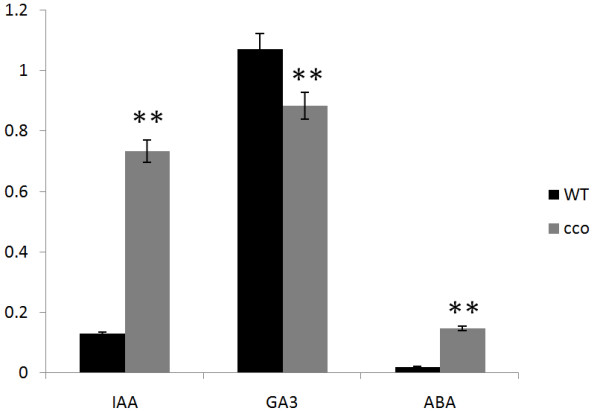
**Quantification of free IAA, GA**_**3**_ **and ABA in WT and the** ***cco*** **seeds at 7 DAF.** **P < 0.01.

The hormone auxin governs a variety of developmental processes [36], including embryogenesis [37]. In embryogenesis, auxin controls cell division and specification, which are critical for establishing the embryo pattern. Most of the pattern formation events in *Arabidopsis* embryogenesis, including cotyledon development, depend on auxin biosynthesis, transport, and response [37]. IAA is the most important natural auxin. Therefore, we examined the endogenous free IAA level in WT and the *cco* mutant seeds at 7 DAF (days after fertilisation) by HPLC analysis (Figure 2). Compared with WT, the *cco* mutant had a higher free IAA level in seeds at 7 DAF.

In addition, the endogenous GA3 and ABA content in WT and *cco* seeds at 7 DAF were quantified. The measurements demonstrated that the ABA content was significantly increased, while the GA3 content was decreased in *cco* (Figure 2). The increased ABA/GA ratio may lead to a low germination rate for the *cco* mutant.

### Auxin sensitivity was depressed in *cco*

**Figure 3:**
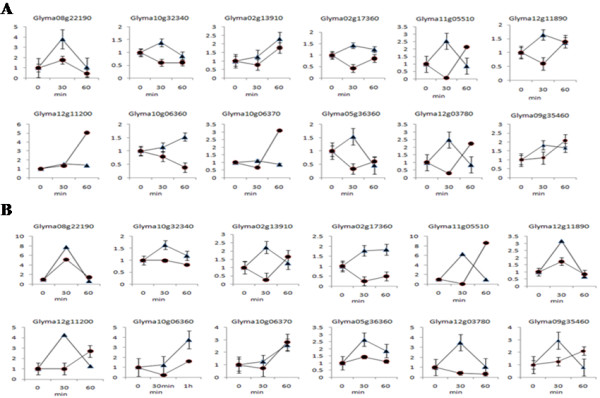
**Regulation of early auxin-regulated genes in WT and the** ***cco*** **muant. (A)** Treatment with 5 × 10^−5^ M 2,4-D. **(B)** Treatment with 2 × 10^−4^ M 2,4-D. WT, triangles; *cco*, circles.

Twelve auxin responsive genes, including two *AUX/IAA* genes (*Glyma08g22190, Glyma10g32340*), five *GH3* genes (*Glyma02g13910, Glyma02g17360, Glyma11g05510, Glyma12g11890, Glyma12g11200*) and five *SAUR* genes (*Glyma10g06360, Glyma10g06370, Glyma05g36360, Glyma12g03780, Glyma09g35460*), were selected to investigate the transcription of early auxin-regulated genes in response to auxin treatment by quantitative RT-PCR (qRT-PCR). As shown in Figure 3, all of these genes were up-regulated in WT at 30 min after treatment with 0.05 μM 2,4-dichlorophenoxyacetic acid (2,4-D). However, in the *cco* mutant, only three genes (*Glyma08g22190, Glyma12g11200, and Glyma09g35460*) were weakly up-regulated, and the other genes had weak variation or even unexpected down-regulation (Figure 3A). When hypocotyls were treated with 0.2 μM 2,4-D, the twelve genes tested showed higher expression levels in WT, and *Glyma08g22190* and *Glyma11g05510* showed greater than five-fold up-regulation (Figure 3B). With the exception of *Glyma12g11890* and *Glyma05g36360*, the expression trends of the genes in *cco* were similar to that observed after treatment with 0.05 μM 2,4-D (Figure 3). Thus, auxin sensitivity were repressed in the *cco* mutant.

### RNA-Seq of WT and the *cco*mutant

**Figure 4:**
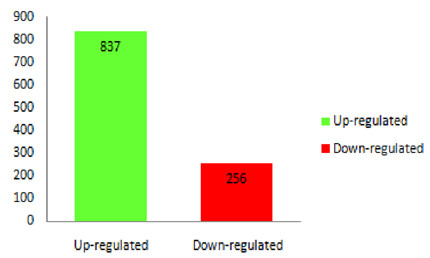
**DEGs between WT and the** ***cco*** **mutant: the up- and down-regulated represent the genes that had an increased and decreased expression levels in** ***cco*** **.**

The positions of the cotyledon primordia in *cco* were generally normal, but the abaxial/adaxial patterning of cotyledons was flawed, which was likely to exist before cotyledon initiation. Thus, we focused on the globular stage, and the pods at 7 DAF were collected for RNA-Seq using the Illumina HiSeq2000 system. A total of 52,870,578 and 50,494,652 successful reads (average length: ~180 bp, approximately 4 × soybean genome) were produced for WT and the *cco* mutant, respectively (Table 1). Notably, more than 85% of the reads mapped back to the soybean reference genome in Phytozome database [38]. In WT, 80.74% of these genes uniquely mapped to a single location, and 80.27% uniquely mapped to a single location for *cco* (Table 1). We identified 39,499 highly confident genes in WT and 39,527 genes in the *cco* mutant. With a False Discovery Rate (FDR) < 0.001 and |log_2_Ratio| ≥ 1, a total of 1,093 genes were differentially expressed; 256 DEGs had lower expression levels in the *cco* mutant, while 837 DEGs were activated (Figure 4 and Additional file 1). In this study, we primarily focused on the differentially expressed genes. The expression levels of the majority of these genes were unaffected in the *cco* mutant, suggesting that *cco* targeted a limited number of genes.

**Table 1:** **Summary of RNA-seq data from WT and** ***cco*** **pods at 7 DAF**

	Total reads	Total base pairs	Total mapped reads	Percent mapped	Unique match	Percent unique match
WT	52,870,578	4,758,352,020	45,064,067	85.23%	42687787	80.74%
*cco*	50,494,652	4,544,518,680	42,914,706	84.99%	40531216	80.27%

**Figure 5:**
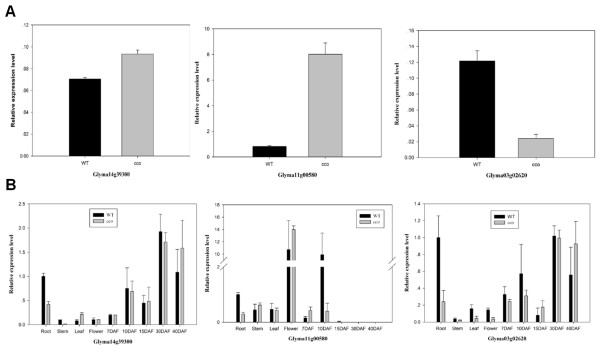
**Confirmation of RNA-seq results using qRT-PCR.** Three DEGs (Glyma14g39300, Glyma11g00580, Glyma03g02620) were selected for confirmation. **(A)** Expression levels of three DEGs in pods at 7 DAF. **(B)** Expression levels of three DEGs in roots, stems, leaves, flowers and seeds at various stages (7, 10, 15, 30 and 40 DAF) of WT and *cco*.

To validate the reliability of the expression profiles obtained using RNA-Seq, we selected fifteen genes with higher or lower expression levels for semi-quantitative reverse transcription PCR (RT-PCR) analysis (Table 2 and Additional file 2: Figure S1A and S1B), and three (*Glyma03g02620, Glyma14g39300, Glyma11g11580*) of the fifteen genes for further qRT-PCR analysis in 7DAF pods from WT and the *cco* mutant (Figure 5A). For all of the genes, the results obtained from the semi-quantitative RT-PCR/qRT-PCR analysis were consistent with data from RNA-Seq.

**Table 2:** **Fold changes of 15 genes selected from RNA-seq data**

Gene ID	Gene description	Fold change	Regulation	P-value
Glyma02g42290	Transmembrane amino acid transporter protein	2.02	Up	0.000139
Glyma03g09140	Transmembrane amino acid transporter protein	2.69	Up	2.58E-07
Glyma05g23180	Membrane transport protein	5.08	Up	3.71E-05
Glyma08g01430	WRKY transcription factor	12.62	Up	3.12E-06
Glyma09g37370	HLH transcription factor	2.35	Down	2.84E-10
Glyma16g13570	bZIP transcription factor	2.03	Up	0.000139
Glyma18g05720	NPH3 family	2.34	Down	1.26E-16
Glyma19g43761	DUF640	4.56	Up	0.000186
Glyma17g14710	YABBY protein	3.05	Up	6.86E-24
Glyma11g00580	tryptophan synthase β-chain	8.01	Up	1.85E-57
Glyma14g39300	ubiquitin-protein ligase activity	371.70	Up	3.14E-08
Glyma03g02620	LBD transcription factor	441.39	Down	8.88E-05
Glyma06g02970	DBB transcription factor	2.91	Up	9.21E-06
Glyma06g17420	HLH transcription factor	2.06	Up	1.74E-68
Glyma16g28310	Fructose-1-6-bisphosphatase	2.16	Down	2.39E-43

In addition, we used semi-quantitative RT-PCR to analyse the expression of four selected genes (*Glyma03g02620, Glyma14g39300, Glyma11g11580, Glyma17g14710*) in WT and *cco* seeds at 7 and 15 DAF (Additional file 2: Figure S1C). Using qRT-PCR, we examined the expression of three genes (*Glyma03g02620, Glyma14g39300, Glyma11g11580*) in the roots, stems, leaves, flowers and seeds at the various developmental stages of WT and *cco*, and the results showed these genes were differentially expressed in these tissues (Figure 5B), indicating some of the DEGs that we obtained were differentially expressed in other tissues.

### Functional classification of differentially expressed genes

**Figure 6:**
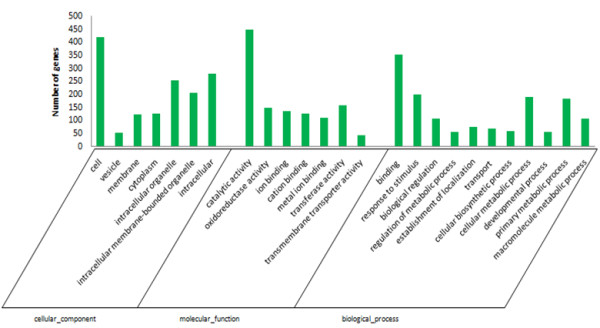
**Gene Ontology classification of the differentially expressed genes between WT and** ***cco.*** Differentially expressed genes are classified into three GO categories: cellular component, molecular function and biological process.

To evaluate the potential functions of the DEGs between WT and the *cco* mutant, Gene Ontology (GO) categories were used to identify key processes for *cco* mutant. Detailed GO term annotations of the DEGs were categorised according to biological processes, molecular functions and cellular components. The GO categories for the set of DEGs (Figure 6) revealed that most of the encoded products were associated with “intracellular organelle”, “catalytic activity”, “response to stimulus” and “cellular metabolic process”.

Biological interpretation of the 650 DEGs was further completed using KEGG pathway analysis. Many of the DEGs were mapped to pathways essential for plant growth and development. Overall, 98 pathways were affected in the *cco* mutant, and some of these pathways were consistent with biological processes previously revealed in the GO analysis. The most frequently represented pathways primarily involved in secondary metabolism, including plant hormone biosynthesis and signal transduction (20.46%), such as auxin, CK, GA, ABA, ET, BR and JA (Additional file 3). Asakura et al. found that genes expressed in the early pod stage of soybean were the most numerous, compared with 2 and 5 mm seeds, and the number of expressed genes gradually decreased with seed development [39]. Commonly expressed genes in these stages accounted for 70–95% of the expressed genes in each stage, and only a small number of genes were differentially expressed, which mainly included β-conglycinin, lipid synthesis, lipoxygenase and seed-maturation proteins [39]. Therefore, most DEGs obtained in this study, including genes that were related to hormone biosynthesis and signalling, could still be identified in the early seed stage. However, some DEGs may have been missed because of tissue-dependent gene expression.

**Figure 7:**
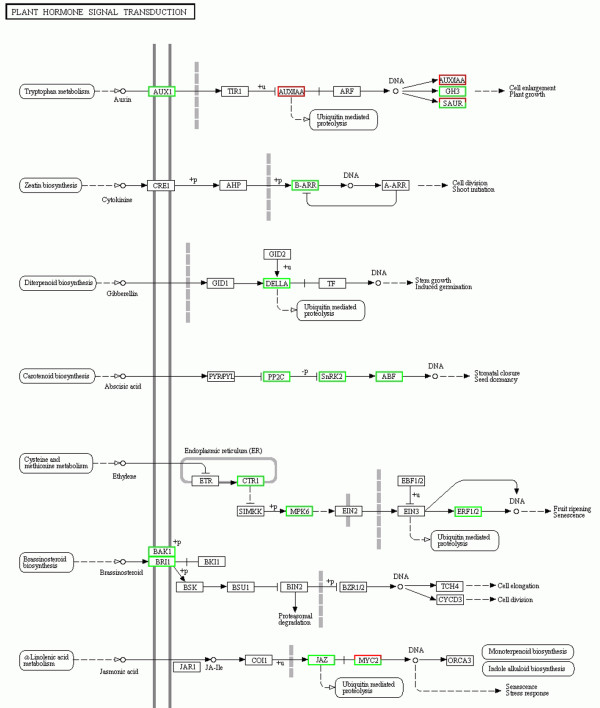
**Transcriptional changes in plant hormone signal transduction pathways.** Differentially expressed genes were mapped to the KEGG pathway database. Genes showing up-regulation and down-regulation are boxed in green and red, respectively.

The results of the transcriptome data showed that many genes encoding proteins involved in auxin metabolism and signal transduction pathways were differentially expressed between *cco* and WT. L-tryptophan, as a primary precursor, is used to generate many indole-containing substances in plants, including IAA. α-subunit anthranilate synthase (ASA) catalyses the conversion of chorismate to anthranilate as one of the first steps in the *Trp* biosynthesis pathway. In *Arabidopsis*, up-regulation of *WEAK ETHYLENE INSENSITIVE2/ANTHRANILATE SYNTHASE α1* (*WEI2/ASA1*) gene through ET results in auxin accumulation in the tips of primary roots [40]. Transgenic rice lines expressing α-SUBUNIT OF ANTHRANILATE SYNTHASE (*OASA1D)* gene show increased production of tryptophan and free IAA [41]. Tryptophan synthase beta subunit (TSB) catalyses the last step in tryptophan biosynthesis. In IAA biosynthesis, indole-3-acetaldoximem (IAOx)-dependent IAA biosynthesis is a specific pathway in plants. In this process, two homologous cytochrome P450 enzymes, CYP79B2 and CYP79B3, mediate the synthesis of IAOx from tryptophan [42], and the double mutation of these two genes results in the downregulation of IAA synthesis. In the transcriptome data obtained in this study, one *ASA* gene, one *TBS* gene and three *CYP79* genes were up-regulated, suggesting that auxin synthesis was enhanced in the *cco* mutant (Additional file 4). Mounting evidence suggests that polar auxin transport (PAT) controls important growth and developmental processes in higher plants. In *Arabidopsis*, studies have shown that the AUXIN1/LIKE-AUX1 (AUX/LAX) family of auxin transporters are major influx carriers, whereas the PIN-FORMED (PIN) family of auxin proteins are major efflux carriers. The AUX/LAX family is represented by four highly conserved genes, namely *AUX1*, *LAX1*, *LAX2*, and *LAX3. LAX2* regulates vascular patterning in cotyledons. Transcriptome data showed that three *LAX2* genes and one *PIN* gene were up-regulated in the *cco* mutant, indicating that auxin polar transport was also influenced. Auxin-responsive genes include *GH3* genes, *small auxin up RNA (SAUR)* genes and *AUX/IAA* genes [43]. In our transcriptome data, one *AUX/IAA* and five *SAUR* genes were down-regulated, and four *SAUR* genes were up-regulated in *cco* (Figure 7). All of the *GH3* genes were up-regulated, which were likely due to the down-regulation of the *AUX/IAA* genes as Aux/lAA proteins repress the expression of *GH3* genes in soybean [44]. Taken together, these data indicated that auxin biosynthesis and signal transduction in *cco* were more active than in WT.

The role of GA in embryo morphogenesis has not yet been determined. In general, GA is required for seed development, which is determined through the analysis of GA-deficient mutants. In pea (*Pisum sativum L.*), the GA-biosynthesis mutant *lh* has provided the best evidence for the physiological role of GA in seed development. The LH protein, which encodes *ent*-kaurene oxidase (KO), is required for the three-step oxidation of *ent*-kaurene to *ent*-kaurenoic acid as an early step in the GA biosynthesis pathway altering seed development [45]. KS (*ent*-kaurene synthase) and KO are key enzymes in the GA biosynthetic pathway, and both are encoded by a single gene in soybean. GA_2_ox (GA_2_-oxidase) plays an important role in the last step of GA biosynthesis and catalyses the inactivation of GAs by conversion to bioactive GAs, thereby affecting the amount of bioactive GAs. Overexpression of *GA*_*2*_*ox* genes results in reduced GA level in *Arabidopsis* [46], rice [47], and poplar [48]. DELLA-domain proteins are transcriptional regulators that repress GA responses, and these proteins are rapidly degraded in response to GA. In this study, one *KO* gene and one *KS* gene were downregulated in the *cco* mutant, suggesting that an early step in GA biosynthesis was blocked (Additional file 5). In addition, four *GA*_*2*_*ox* genes and three *DELLA* genes were up-regulated, suggesting that the rate of conversion of inactive GAs to bioactive GAs was limited for the *cco* mutant (Figure 7 and Additional file 5).

ABA not only plays an important role in the stress responses and plant tolerance, but also controls seed development and germination. The enzyme 9-cis-epoxycarotenoid dioxygenase (NCED) is involved in a rate-limiting step for ABA biosynthesis. Recently, Martínez-Andújar et al. reported that the *NCED6* induction enhanced seed dormancy in *Arabidopsis* [49]. Here, we showed that *NCED* genes were up-regulated in the *cco* mutant, suggesting that ABA synthesis was promoted. However, another gene encoding abscisic acid 8′-hydroxylase, which catalyses a key step in ABA catabolism, was also up-regulated (Additional file 6). Snf1-related protein kinases (SnRK2s) are key positive regulators in ABA signal transduction pathway. In the absence of ABA, protein phosphatases type 2C (PP2Cs) physically interact with SnRK2s and negatively regulate ABA responses. However, interactions between PP2Cs and SnRK2s are disrupted when an ABA molecule binds to PYRABACTIN RESISTANCE1 (PYR1)/PYR1-LIKE (PYL)/REGULATORY COMPONENTS OF ABA RECEPTORS (RCAR) receptors, leading to structural changes in these receptors. When SnRK2s are released from PP2C inhibition, their downstream targets, including ABA-Responsive Element Binding Factors (ABFs), are activated [50]. Fujii et al. constructed an *Arabidopsis* line carrying mutations in all 10 members of the SnRK2 family. The decuple mutant *snrk2.1/2/3/4/5/6/7/8/9/10* (*srk2g/d/e/a/h/e/f/c/j/b*) was defective in gene regulation and ABA accumulation [51]. In our data, the *PP2Cs*, *SnRK2* and *ABF* genes were up-regulated in the *cco* mutant (Figure 7). In summary, ABA biosynthesis, catabolism and signalling were enhanced in *cco*.

In addition to auxin, GA and ABA, the pathways of other hormone biosynthesis and signal transduction (such as CK, ET, BR, and JA) were also influenced in the *cco* mutant. CK and ET, as major classes of plant hormone, are involved in various aspects of plant development, including organ development [52, 53]. In *cco,* we found two genes encoding adenosine-phosphate isopentenyltransferase (IPT), a rate-limiting enzyme in CK biosynthesis, expressed at significantly higher levels [54, 55] (Additional file 7). In CK signalling, type-B ARABIDOPSIS RESPONSE REGULATORS (ARRs) are transcriptional activators that regulate cytokinin targets, including type-A ARRs [56]. We observed the up-regulation of two type-B ARR genes (Figure 7). Aminocyclopropane-carboxylate oxidase (ACO), an “ethylene-forming enzyme” (EFE), catalyses the final step in ethylene biosynthesis, converting l-amino-cyclopropane-1-carboxylic acid (ACC) into ethylene, CO2, and cyanide. Four *ACO* genes were up-regulated in *cco* (Additional file 8). Antisense constructs of *ACO* genes can notably reduce ET production [57]. ET signalling was also influenced in the *cco* mutant, and the expression levels of ethylene-related genes encoding serine/threonine-protein kinase (CTR1), mitogen-activated protein kinase 6 (MPK6), and ethylene-responsive transcription factor (ERF1/2) were altered in *cco* (Figure 7 and Additional file 8). BRs regulate embryonic and post-embryonic development, adult homeostasis and expression of numerous genes [58]. Plant steroid hormones are recognised by brassinosteroid-insensitive 1 (BRI1), whose kinase activity is activated by exogenous BR application [59]. We also found that the expression levels of thirteen *BR1* and four *BRI1 Associated receptor Kinase 1 (BAK1)* genes in *cco* were higher than those in WT (Figure 7). JA regulates plant growth and stress responses, biosynthetic pathway of which starts with α-linolenic acid [60]. The DEGs in JA biosynthesis and signalling pathway included those that encode phospholipase A1, 12-oxo-phytodienoic acid reductase, lipoxygenase, JASMONATE-ZIM (JAZ) and MYC2 proteins [61] ( Additional file 9 and Figure 7).

Auxin, BR, CK and GA are generally considered major developmental growth regulators, whereas ABA, ET and JA are involved in stress responses, all of these hormones converge on auxin [62, 63]. Auxin is not only a hormone but also a morphogen. Auxin accumulation is controlled by auxin biosynthesis and metabolism, the conjugation/deconjugation of active auxin to/from its inactive conjugated form and auxin transport [64–66]. Auxin maxima and activity have been implicated in embryo development and patterning [37]. In *cco*, the free IAA level and auxin polar transport were affected. Polar auxin transport promotes the formation of local auxin maxima and gradients within tissues that play important roles in auxin action, such as the patterns of cell division and differentiation in the root meristem [67] and the establishment of cotyledons [26, 29]. Plants show directional growth in response to environmental stimuli such as light or gravity (tropisms). It was found that auxin played an important role in tropisms [68] and unequal auxin distribution across organs may lead their bending [69]. The cotyledons of *cco* showed outward bending, which allowed us to hypothesise that PAT might cause an auxin imbalance between the abaxial and adaxial side in cotyledons of the *cco* mutant, resulting in the adaxial side developing faster than the abaxial side. However, in soybean embryogenesis, the endosperm surrounds the embryo and cotyledons, the curled cotyledons observed in the *cco* mutant might reflect the fetter of the endosperm. Many signals can regulate auxin polar transport including stress, protein phosphorylation, reactive oxygen, flavonols and other hormones [70–73], which among these signals affect(s) PAT in *cco* require further investigation. In *cco*, only the shape of cotyledons not including leaves was abnormal, indicating that signal(s) or mutated genes regulating PAT were specific for cotyledon initiation and growth.

GA and ABA have mainly been implicated in later stages of embryo development, acting antagonistically in the regulation of seed germination [74]. Compared with WT, *cco* showed higher IAA and ABA levels, whereas a lower GA_3_ level. Several reports indicate that auxin and GA overlap in the regulation of multiple aspects of plant development, and there is positive cross-talk interaction between them. Willige et al. have reported that a mutant deficient in GA biosynthesis and signalling showed a reduced PIN protein level, which could be recovered by exogenous GA application. Moreover, GA could interact with PIN1 and promote cotyledon differentiation [75]. Auxin can also induce GA biosynthesis. Root cells with lower auxin level have reduced GA biosynthesis [76, 77]. Interestingly, our results demonstrated a lower GA_3_ but higher IAA level in *cco* seeds. But beyond that, *cco* seeds had higher ABA content, which might correlate with lower GA_3_ level and may be the reason for lower germination rate in *cco*. Consistently, ABA negatively regulates bioactive GA level during seed germination.

## Conclusions

Multiple phytohormone biosynthesis and signalling pathways are reprogrammed in the *cco* mutant, which is consistent with our HPLC analysis. Our results indicate that curled-cotyledons and reduced seed germination rate may result from the biosynthesis and signalling disorder of multiple phytohormones. Accumulating evidence supports the idea that hormones not only act in a linear pathway but also function in a complex network of feedback regulation and cross talk within or between different hormone pathways. At present, the studies of molecular mechanisms underlying hormonal cross talk mainly focus on postembryonic development, including the roots, shoots and leaves. However, adequate models of the hormonal network involved in cotyledon development are not well established in plants. Analysis of *cco* may provide a promising frame for further studies of hormone interactions and offer the potential discovery of new genes controlling cotyledon development in plants.

## Methods

### Plant materials

Soybean seeds from wild type cultivars Nannong 94–16 and its cotyledon mutant *cco* were provided by Soybean Research Institute, Nanjing Agricultural University, China. The seeds were cultivated in field under natural conditions in Nanjing, China. Total RNA was extracted from the WT and *cco* tissues, including the roots, stems, leaves, flowers and seeds, which were frozen in liquid nitrogen and stored at −80°C until further analysis.

### Plant paraffin section

For paraffin sectioning, wild type and mutant seeds at various developmental stages were fixed in Carnoy’s fluid (ethanol/glacial acetic acid, 3:1) at room temperature followed by dehydration in a graded ethanol series, staining with 1% haematoxylin solution, and clearance with xylene. The seeds were embedded in paraffin and sectioned at 10 to 15 μm (Leica, RM2135).

### Semi-quantitative RT-PCR and quantitative RT-PCR (qRT-PCR)

To analyse the gene expression in soybean, total RNA was extracted with the Plant RNA Extract Kit (TianGen, Beijing, China) according to the manufacturer’s instructions. PCR amplification was performed with PrimeScript 1^st^ Strand cDNA Synthesis Kit (Takara). qRT-PCR was performed as described previously [78]. The *Gmtubulin* (GenBank no.AY907703) and *Gmactin* (GenBank no.XM_003531354) genes were used as reference genes for qRT-PCR and semi-quantitative RT-PCR, respectively. The primers used in this study were provided in Additional file 10.

### Extraction of endogenous free IAA, GA_3_, ABA and HPLC analysis

A total of 0.5 g fresh seeds at 7DAF from at least 10 individual lines of WT and *cco* were ground in liquid nitrogen, phytohormones were extracted in 10 milliliter cold 80% (v/v) methanol and 1% (w/v) polyvinylpyrrolidone was added as an antioxidant. After 24 h of incubation at 4°C, the samples were centrifuged for 15 min at 12,000 g at 4°C. The supernatant was retained, and the pellet was re-extracted with 10 ml of cold 80% (v/v) methanol and recentrifuged as indicated above. The supernatants were pooled. After evaporation under a N_2_ gas flow, the pH of the remaining water phase was adjusted to 3.0. IAA, GA_3_ and ABA purification was performed using acetyl acetate. The acetyl acetate was also evaporated under an N_2_ stream. The resulting dried precipitate was collected in 0.5 ml of 100% methanol, filtered through 0.45 μm filter membrane and submitted to HPLC (Shimadzu, Kyoto, Japan) analysis. HPLC analysis was performed using an Alltima C18 column (4.6 mm × 250 mm). The IAA and ABA concentration was determined at 254 nm, and the GA_3_ concentration was determined at 210 nm. Hormone measurements were conducted in triplicate and subjected to statistical analysis.

### IAA sensibility assay

WT and *cco* seeds were germinated in moistened vermiculite at 30°C. After 72 h, the seedlings were sprayed with 2.5 × 10^−3^ M 2,4-D solution (pH 6). The intact hypocotyls were then segregated from the seedlings and incubated in 10 ml of incubation medium in the absence or presence of 5 × 10^−5^ M and 2 × 10^−4^ M 2,4-D (pH 6) at 30°C with continuous shaking. At 0.5 and 1 h, the 1.2-cm sections obtained from the elongating region of the hypocotyls (0.5-1.7 cm below the cotyledons) were excised. The hypocotyl sections were rinsed in deionised distilled H_2_0 and immediately frozen in liquid N_2_. The sections were stored at −70°C until further use for RNA extraction.

### cDNA library construction for RNA-Seq and sequencing

Beads with oligo (dT) were used to isolate poly (A) mRNA after total RNA was collected. Fragmentation buffer was added to digest the mRNA to generate short fragments. These short fragments were used as templates, and random hexamer-primers were used to synthesise first-strand cDNA. Second-strand cDNA was synthesised in a reaction containing buffer, dNTPs, RNase H and DNA polymerase I. Short fragments were purified using the QIAquick PCR extraction kit (QIAGEN) and eluted in EB buffer for end preparation and poly (A) addition. Subsequently, the short fragments were ligated with sequencing adaptors. For PCR amplification, we selected suitable fragments to use as templates based on the results of agarose gel electrophoresis. The libraries were sequenced using the Illumina HiSeq™ 2000 system.

### Raw read filtering and mapping to the reference genome and genes sequences

Dirty raw reads were discarded via the following four steps: 1. reads with adaptors were removed; 2. reads with unknown nucleotides larger than 5% were removed; 3. reads with low quality (more than half of the base quality less than 5) were removed; 4. clean reads were obtained. Clean reads were mapped to the reference genome and genes sequences respectively using SOAPaligner/soap2. Mismatches with no more than m (m default is 5) bases were used in the alignment. All subsequent analyses were based on clean reads.

### Gene expression annotation

Gene coverage is the percentage of a gene covered by reads. This value equals the ratio of the number of bases in a gene covered by unique mapping reads to the number of total bases in that gene. The UniGene expression was calculated using the RPKM method (Reads Per kb per Million reads). The RPKM method eliminated the influence of different gene lengths and sequencing discrepancies on the gene expression calculations. Therefore, the calculated gene expression could be used to directly compare differences in gene expression between the samples. If there was more than one transcript for a gene, the longest transcript was used to calculate the expression level and coverage.

### Differentially expressed gene analysis

Using “The significance of digital gene expression profiles” [79], we identified differentially expressed genes between WT and the *cco* mutant based on the following criteria: (FDR) < 0.001 and |log_2_Ratio| ≥ 1.

### GO enrichment and KEGG pathway analysis

The differentially expressed genes were subjected to GO enrichment analysis. The analysis first mapped all DEGs to GO terms in the database (http://www.geneontology.org/), calculating gene numbers for every term, followed by the ultra-geometric test to identify significantly enriched GO terms in DEGs compared with the genome background. KEGG Pathway (http://www.genome.jp/kegg/pathway.html) enrichment analysis was used to identify significantly enriched metabolic or signal transduction pathways in DEGs compared with the whole genome background.

## Availability of supporting data

The RNA-seq data of this article have been deposited in the Gene Expression Omnibus (GEO) Database, with the following access number: GSE58354 (http://www.ncbi.nlm.nih.gov/geo/query/acc.cgi?acc=GSE58354). Other supporting data are included as additional files.

## Electronic supplementary material

Additional file 1: **1093 differentially expressed genes between WT and** ***cco*** **.** (XLS 91 KB)

Additional file 2: **Semi-quantitative RT-PCR analysis of 15 selected genes. (A)** and **(B)**: semi-quantitative RT-PCR analysis of 15 selected genes in WT and *cco* pods at 7 DAF. **(C)**: semi-quantitative RT-PCR analysis of 4 of the 15 selected genes in WT and *cco* seeds at 7 and 15 DAF. 1: WT seeds at 7 DAF; 2: *cco* seeds at 7 DAF; 3: WT seeds at 15 DAF; 4: *cco* seeds at 15 DAF. (TIFF 1 MB)

Additional file 3: **Differentially expressed genes in hormone biosynthesis and signal transduction pathways.** (XLSX 117 KB)

Additional file 4: **Transcriptional changes in tryptophan metabolism.** Differentially expressed genes from RNA-Seq were mapped to the KEGG pathway database. The up-regulated genes in *cco* are boxed in green. (TIFF 4 MB)

Additional file 5: **Transcriptional changes in zeatin biosynthesis.** The up-regulated and down-regulated genes in *cco* are boxed in green and red, respectively. (TIFF 4 MB)

Additional file 6: **Transcriptional changes in diterpenoid biosynthesis.** The up-regulated and down-regulated genes in *cco* are boxed in green and red, respectively. (TIFF 5 MB)

Additional file 7: **Transcriptional changes in carotenoid biosynthesis pathway.** The up-regulated and down-regulated genes in *cco* are boxed in green and red, respectively. (TIFF 4 MB)

Additional file 8: **Transcriptional changes in cysteine and methionine metabolism pathway.** The up-regulated and down-regulated genes in *cco* are boxed in green and red, respectively. (TIFF 4 MB)

Additional file 9: **Transcriptional changes in α-linolenic acid metabolism pathway.** The up-regulated and down-regulated genes in *cco* are boxed in green and red, respectively. (TIFF 4 MB)

Additional file 10: **The list of primers used in this article.** (XLSX 12 KB)
