# Supplementary figures and images for: RNA-Seq analysis reveals that multiple phytohormone biosynthesis and signal transduction pathways are reprogrammed in curled-cotyledons mutant of soybean [Glycine max (L.) Merr.]
Source: BMC Genomics. 2014 Jun 21;15(1):510. doi: 10.1186/1471-2164-15-510 (PMC4078243; doi:10.1186/1471-2164-15-510)

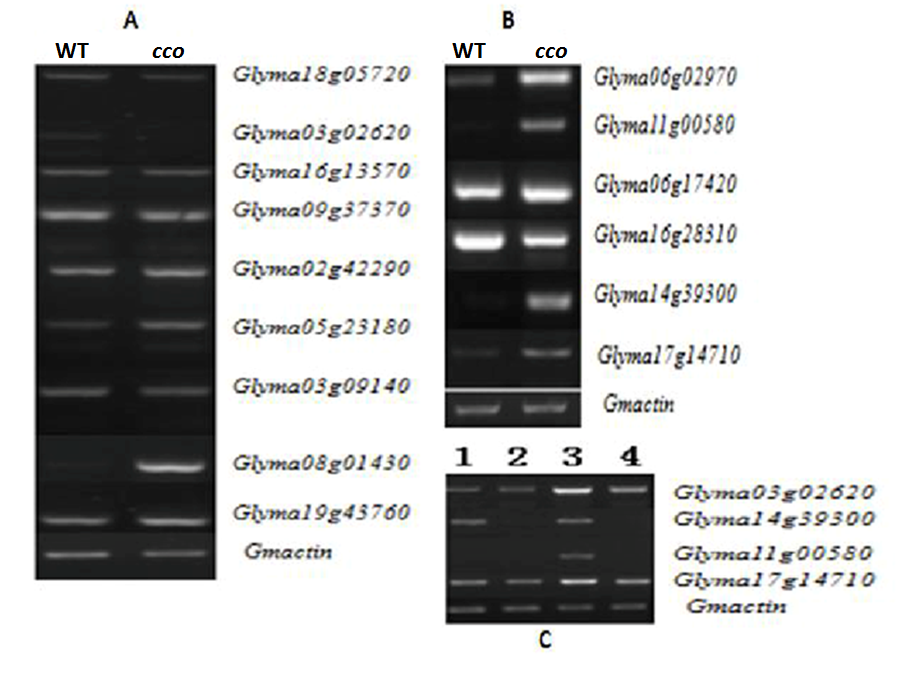

Supplement: Supplementary file 2 — Additional file 2: Semi-quantitative RT-PCR analysis of 15 selected genes. (A) and (B): semi-quantitative RT-PCR analysis of 15 selected genes in WT and cco pods at 7 DAF. (C): semi-quantitative RT-PCR analysis of 4 of the 15 selected genes in WT and cco seeds at 7 and 15 DAF. 1: WT seeds at 7 DAF; 2: cco seeds at 7 DAF; 3: WT seeds at 15 DAF; 4: cco seeds at 15 DAF. (TIFF 1 MB) [file 12864_2014_6175_MOESM2_ESM.tiff]

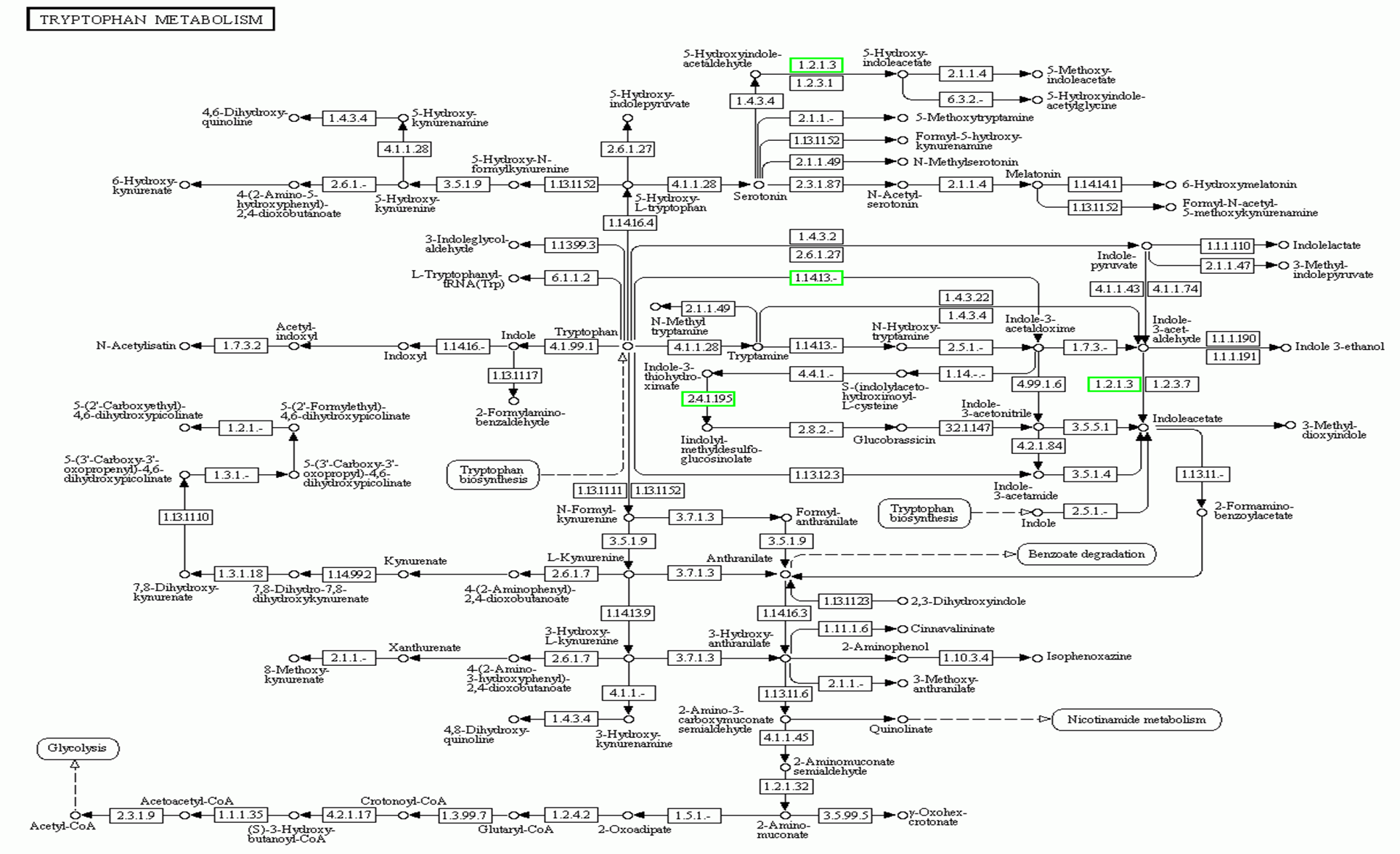

Supplement: Supplementary file 4 — Additional file 4: Transcriptional changes in tryptophan metabolism. Differentially expressed genes from RNA-Seq were mapped to the KEGG pathway database. The up-regulated genes in cco are boxed in green. (TIFF 4 MB) [file 12864_2014_6175_MOESM4_ESM.tiff]

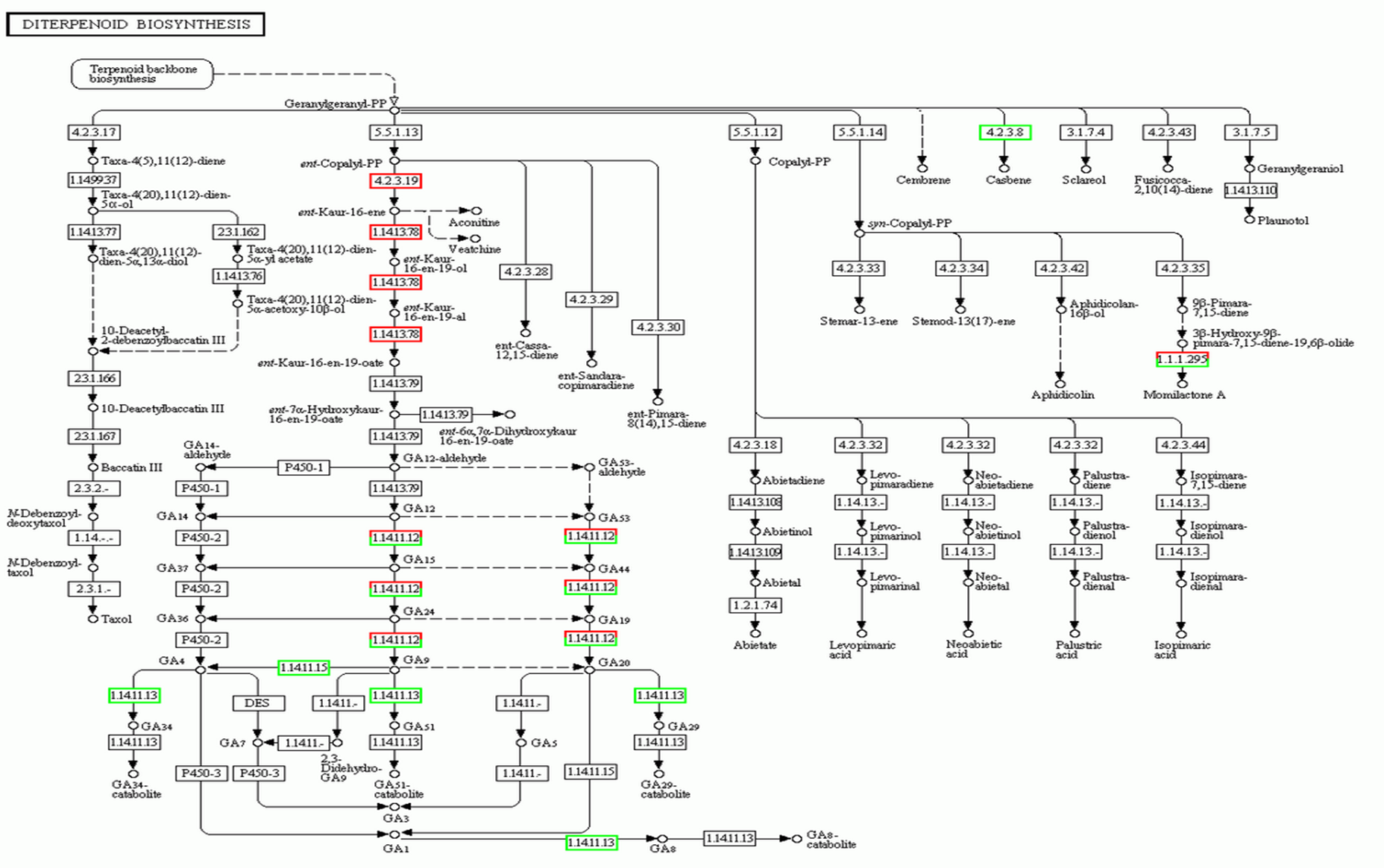

Supplement: Supplementary file 5 — Additional file 5: Transcriptional changes in zeatin biosynthesis. The up-regulated and down-regulated genes in cco are boxed in green and red, respectively. (TIFF 4 MB) [file 12864_2014_6175_MOESM5_ESM.tiff]

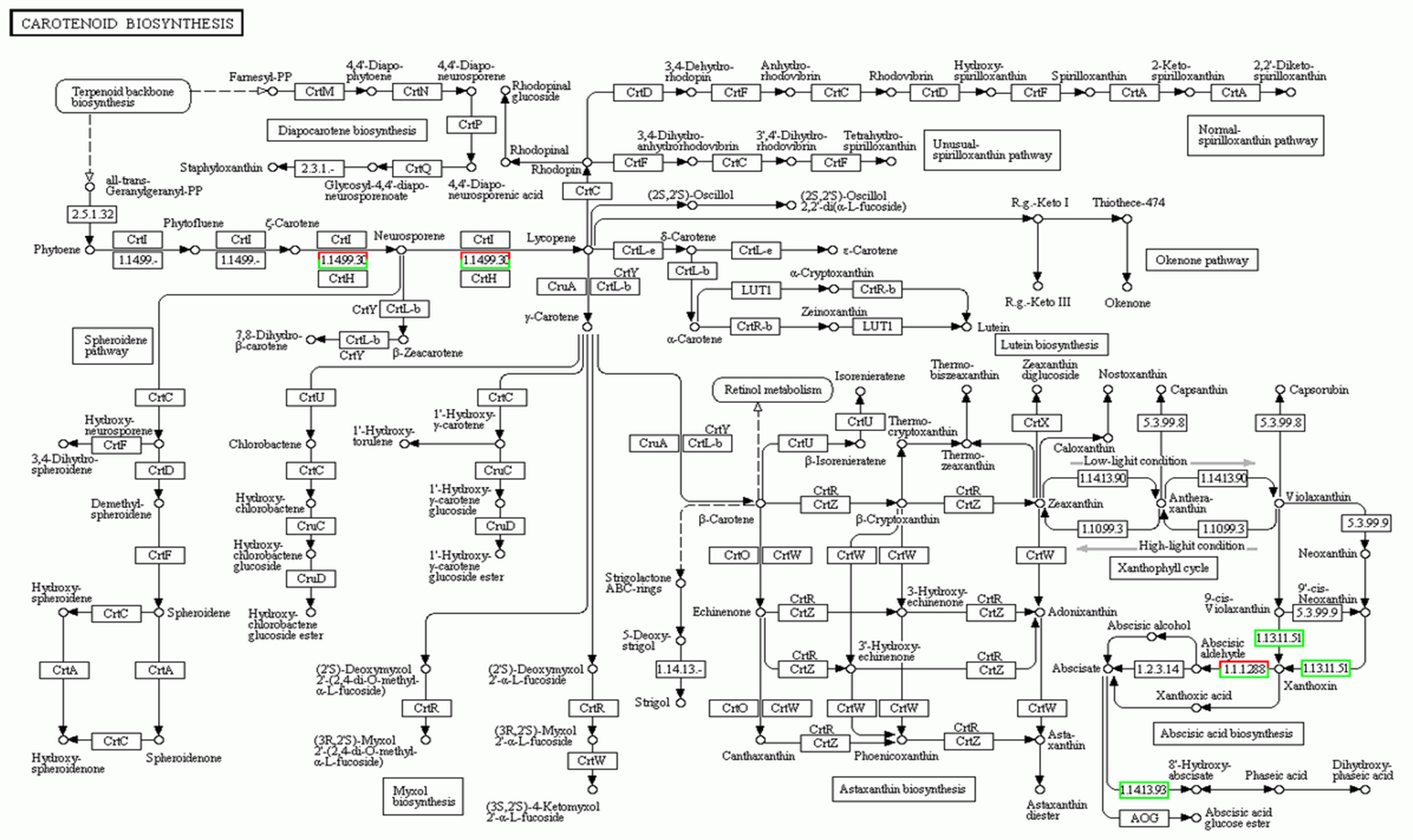

Supplement: Supplementary file 6 — Additional file 6: Transcriptional changes in diterpenoid biosynthesis. The up-regulated and down-regulated genes in cco are boxed in green and red, respectively. (TIFF 5 MB) [file 12864_2014_6175_MOESM6_ESM.tiff]

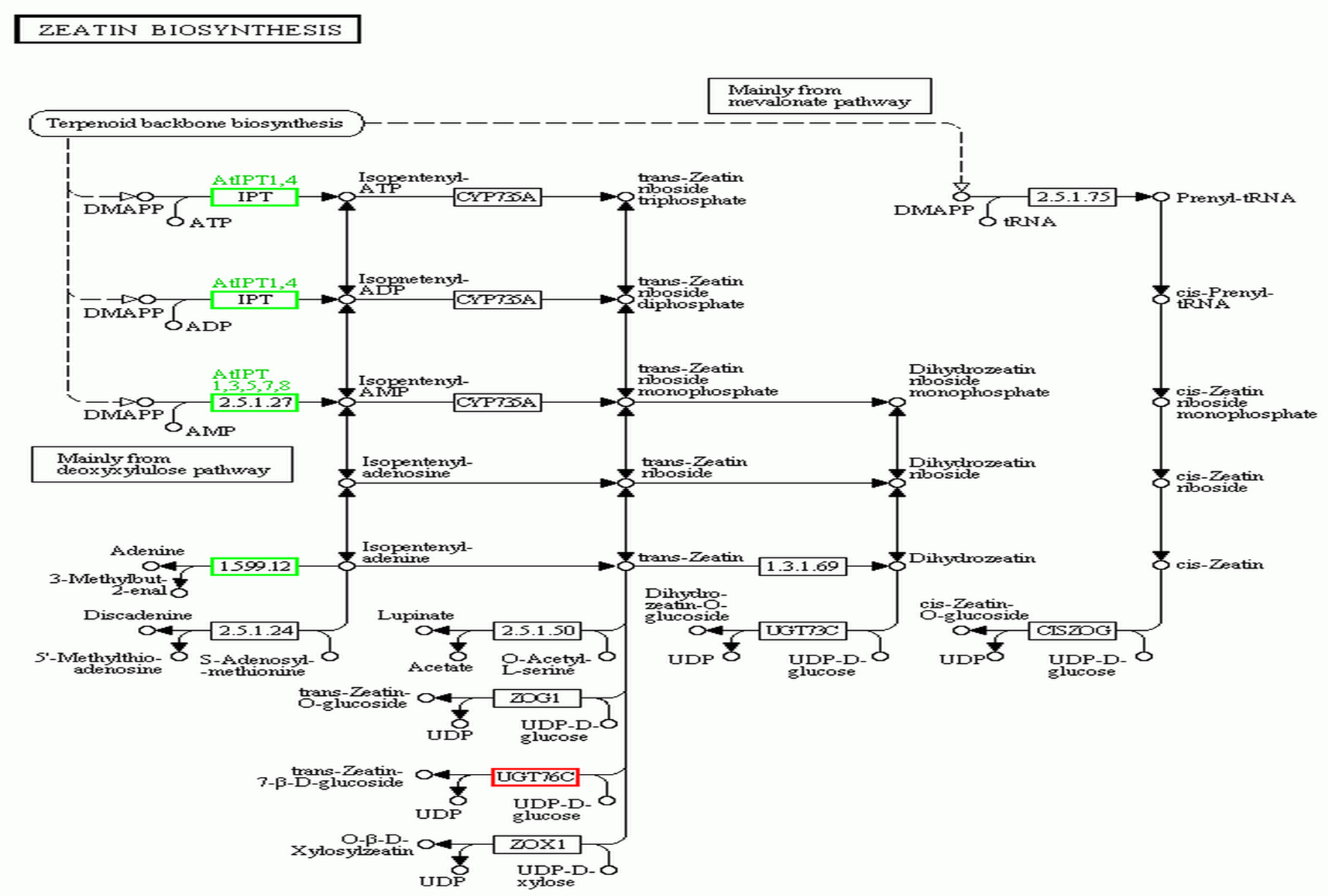

Supplement: Supplementary file 7 — Additional file 7: Transcriptional changes in carotenoid biosynthesis pathway. The up-regulated and down-regulated genes in cco are boxed in green and red, respectively. (TIFF 4 MB) [file 12864_2014_6175_MOESM7_ESM.tiff]

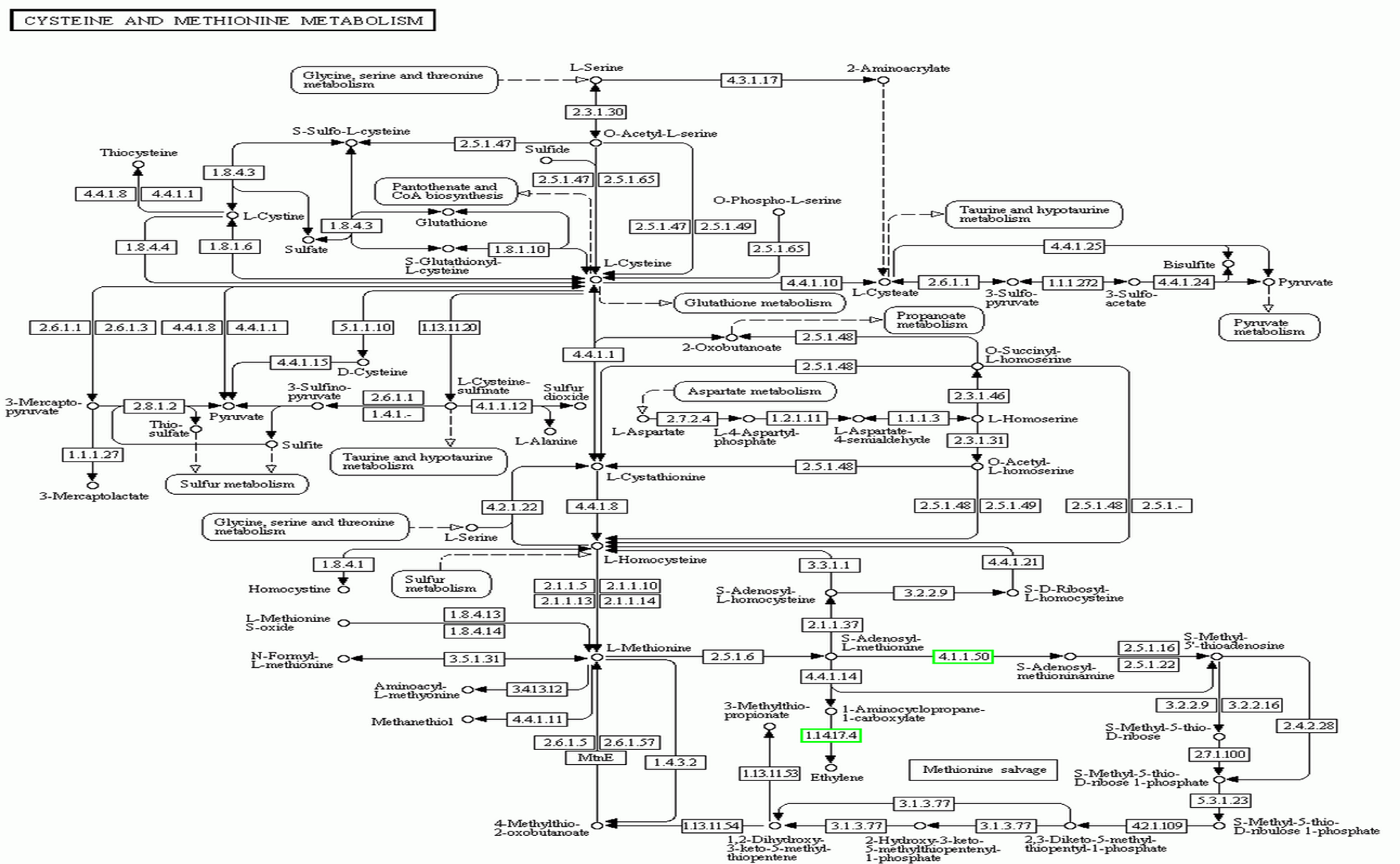

Supplement: Supplementary file 8 — Additional file 8: Transcriptional changes in cysteine and methionine metabolism pathway. The up-regulated and down-regulated genes in cco are boxed in green and red, respectively. (TIFF 4 MB) [file 12864_2014_6175_MOESM8_ESM.tiff]

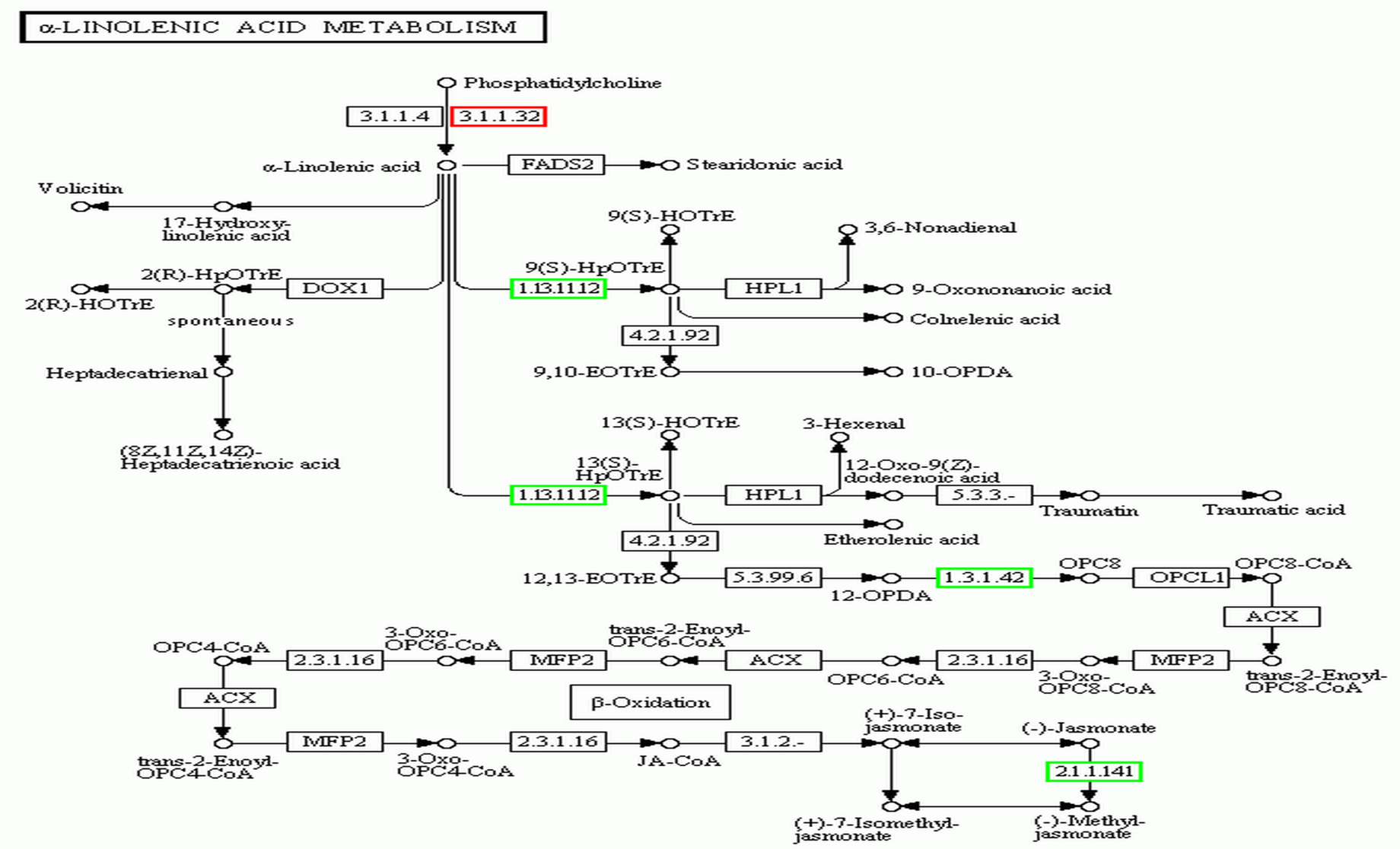

Supplement: Supplementary file 9 — Additional file 9: Transcriptional changes in α-linolenic acid metabolism pathway. The up-regulated and down-regulated genes in cco are boxed in green and red, respectively. (TIFF 4 MB) [file 12864_2014_6175_MOESM9_ESM.tiff]
